# “The patients first and foremost” collaborative practice in the Australian healthcare system: a qualitative study

**DOI:** 10.12688/mep.20512.2

**Published:** 2025-05-07

**Authors:** Sarah Meiklejohn, Lynda Cardiff, Bronwyn Clark, Brian Jolly, Josephine Maundu, Theanne Walters, Glenys Wilkinson, Fiona Kent

**Affiliations:** 1 Faculty of Medicine, Nursing and Health Sciences, Monash University, Clayton, Victoria, Australia; 2 Australian Pharmacy Council, Canberra Airport, Australian Capital Territory, Australia; 3 School of Medicine & Public Health, College of Health, Medicine and Wellbeing, The University of Newcastle, Callaghan, New South Wales, Australia; 4 Australian Medical Council, Kingston, Australian Capital Territory, Australia; 5 Royal College of Surgeons in Ireland, Dublin, Ireland

**Keywords:** collaborative practice, interprofessional education, health professions education, medical education, health practitioners, consumer, patient centred care, accreditation authorities

## Abstract

**Background:**

Collaborative patient centred practice is an expectation of the Australian healthcare system, yet there is not a clear understanding of what this entails. The aim of this research was to describe collaborative practice, as understood within the context of the Australian health system.

**Methods:**

Nineteen focus groups were conducted in 2022 with 84 participants consisting of education providers (n=62), consumers (n=10), representatives from the Health Profession’s Education Standing Group (n=8), and health service practitioners (n=4). Framework analysis was undertaken to understand facilitators of, and barriers to, collaborative practice and learning within the Australian education and healthcare systems.

**Results:**

Participants were asked to describe examples of effective collaborative practice, what they would expect to experience, and examples of when collaborative practice did not occur and the associated outcomes. Participants from all groups emphasised the importance of elevating the patient voice within a patient centred collaborative healthcare team. Patients, family and carers needed to be positioned as central team members within a collaborative healthcare team. Power and hierarchy within the healthcare team impacted on the ability to deliver collaborative practice.

**Conclusions:**

By positioning the patient and their family and carers as members of the team, shared goals for optimal patient outcomes were experienced. By contrast when collaborative practice did not occur, or patients were omitted as central team members, poor communication and disjointed healthcare was described, leaving patients feeling disempowered and disengaged.

## Introduction

Contemporary healthcare features the active participation of patients and their support network. In contrast to the traditional paternalistic approach, current expectations are for shared decision-making and greater patient autonomy, often supported by health policy and research ( Towle *et al.*, 2016). To facilitate increased patient involvement, a modification of traditional models of care is required. In particular, the acceptance that patients can make valid decisions, supported by the professional guidance of healthcare providers ( Chin, 2002). Interprofessional collaborative practice describes this way of working “when multiple health workers from different professional backgrounds work together with patients, families, carers and communities to deliver the highest quality of care” ( WHO, 2010). In order to prepare health professionals to be able to provide collaborative practice, educators designing health profession education programs require a comprehensive understanding of patient expectations. The benefits of involving patients in the education of health professionals is supported by literature ( Towle *et al.*, 2010), however patient expectations regarding collaborative care are less clear.

In some settings, healthcare is provided by interprofessional which has been associated with improved patient outcomes ( Madsen *et al.*, 2022; Reeves *et al.*, 2013; Schentrup *et al.*, 2019). Patients in a primary care setting receiving a collaborative model of care report positive experiences including improved relationships with healthcare providers and a greater feeling of involvement in the wider care team ( Davidson *et al.*, 2022; Morgan *et al.*, 2020). A positive relationship has also been identified between patient experience and healthcare outcomes according to both self-rated and objectively measured indicators ( Doyle *et al.*, 2013) providing further incentive to increase the patient healthcare experience. Finally, improvements in quantitative patient outcomes can be achieved through targeted interprofessional learning interventions by healthcare teams ( Webster *et al.*, 2024).

This research is part of a larger study that sought to explore the views of collaborative practice from multiple stakeholders, and to use these views to shape accreditation authority practices to contribute to the development of collaborative health professionals ( HPAC Forum, 2022).

## Methods

### Study design

A qualitative research design was undertaken underpinned by a social constructivist and interpretivist theoretical stance. Social constructivism recognises that there is no single reality, that knowledge is constructed from experience and therefore there is value in exploring multiple perspectives ( Crotty, 1998). The perspective of health consumers, education providers and health practitioners were sought to describe collaborative practice in the Australian healthcare system. Online focus groups were conducted with each stakeholder group separately, and the questions modified slightly in wording for each group.

### Participant recruitment

Recruitment of all stakeholders was undertaken by approaching appropriate external organisations by email. Health consumers were recruited via the Consumers Health Forum and Health Care Consumers Australia. Education providers were recruited via the health profession accreditation authorities represented in the Health Professions Accreditation Collaborative Forum ( HPAC Forum, 2022). Health service providers were contacted via state government health service leaders, professional organisations, and stakeholder networks. Eligibility criteria were developed for each participant group and provided to stakeholder organisations who recruited participants using various methods including direct email, social media or newsletters in September 2022. A webpage was also created to provide information about the research study for potential participants.

Potential participants from all stakeholder groups were asked to submit an online expression of interest. The explanatory statement and consent process was then communicated. All participants provided written consent prior to attending the focus group session. Ethics approval was granted by the Monash University Human Research Ethics Committee (ID: 34594, approved 31/10/2022).

### Data collection

Focus groups were facilitated by one of four members of the research team (FK, LC, JG, JM). A second team member took notes. A semi-structured interview guide informed the questions. All focus groups commenced with a description of research aims and questions, introduction to a shared definition of collaborative practice, followed by semi-structured questions. Focus group questions focused on participant perspectives and experience of collaborative practice (either as a consumer, practitioner in a healthcare team, or representative of an organisation/health system). Examples of ineffective collaborative practice and associated impacts were also sought.

Focus groups were conducted via Zoom videoconferencing between October and November 2022. Interviews lasted between 60 and 90 minutes and were audio recorded and transcribed verbatim using a third-party transcription service.

### Data analysis

Transcripts were verified by two members of the research team. Names and locations were removed from the transcripts to preserve anonymity. Transcribed interviews were loaded into NVivo 20 (QSR International) for framework analysis.

Framework analysis provided a focused yet flexible and repeatable procedure and includes five steps: 1) familiarisation, 2) identifying a coding framework, 3) indexing, 4) charting and 5) mapping and interpretation ( Ritchie & Spencer, 1994). Familiarisation was undertaken using an inductive coding process. All research team members coded a sample of transcripts independently initially, before gathering as a group to propose an initial coding framework. Transcripts were then coded against the initial framework. After an additional meeting to discuss common themes and interpretations, two members of the team finalised the coding framework (S.M, F.K) before coding all transcripts. The remaining steps of charting, mapping and interpretation were undertaken using NVivo, handwritten notes and mind maps.

Two members of the research team met on multiple occasions to discuss interpretations of the findings in relation to the research aims. These interpretations were discussed on several occasions with the broader research team, until consensus was reached. During the analysis, quotes that emphasised or summarised important findings were recorded to highlight dominant themes. For the reporting of this this study we elected to use the term ‘patient centred care’ as this was the language typically used by our consumer representatives. It is however recognised that the term ‘person-centred care’ and other alternatives have been adopted as a preferred term elsewhere ( CIHC, 2024).

### Rigour and trustworthiness

Careful consideration was given to the alignment of aims, methodology and methods used in this study to ensure credibility as a key step for ensuring rigour within health professions education research. Dependability was achieved with interview guides for each focus group. An interprofessional and collaborative lens was applied through the research process through inclusion of the entire research team in the coding process with all interview transcripts cross-checked by at least two members of the research team ( Stenfors *et al.*, 2020). At least two members of the research team attended each focus groups. The research team hold professional backgrounds in medicine, nutrition and dietetics, pharmacy and physiotherapy and education. Educational research PhDs are held by SM, BJ, LC, JG, FK, while BC, BJ, GW, JG, JM hold professional leadership and accreditation roles

## Results

Nineteen (n=19) focus groups involving eighty-four (n=84) participants were conducted.

This included two consumer focus groups (n=10 participants), fourteen education provider focus groups (n=62 participants), two health practitioner focus groups (n = 4 participants), and one focus group was comprised of representatives from the Health Professions Education Steering Group, of Universities Australia a national peak body representing 39 Australian universities (n=8 participants). Education providers self-identified across nineteen (n=19) different health professions, where some education providers represented health professions programs other than their own professional background. Education providers were representative of fourteen (n=14) of the fifteen health professions represented under the Australian National Registration and Accreditation Scheme ( Australian Government Department of Health and Aged Care, 2023). Considered collectively, the research includes perspectives from the following health profession education programs: Aboriginal and Torres Strait Islander Health Practice, chiropractic, dental, medicine, medical radiation practice, nursing, midwifery, optometry, osteopathy, occupational therapy, paramedicine, pharmacy, physiotherapy, podiatry, and psychology.

Thirteen (n=13) education providers included in the focus groups did not represent one single health profession. Several education providers described roles that incorporated several health professions or roles representing self-regulated professions including dietetics and speech pathology.

Detailed examples and experience of both ineffective and effective collaborative practice were captured. The themes and relevant sub-themes are detailed below.

### Patient positioning within ineffective collaborative practice

There were multiple narratives describing ineffective collaborative practice. Such narratives demonstrated an imbalance in patient positioning, with the health professional perspective dominant and the patient perspective disregarded, which contributed to poor communication between consumers with their healthcare team. This resulted in consumers having to either attempt to advocate for themselves and/or duplication of tasks.

*So they (the dermatology team) were the only ones who really needed to be involved and I would have a specialist appointment with an immunologist in the coming months anyway. And that's when I said, “Do you not think this should be collaborative? Are you interested in doing a collaborative practice here?” And that's when I got the death stare almost like don't even attempt that, we're not interested. Consequently, that meant that the next time I went to see the immunologist, I had to fill them in on what my daughter was on. Also, when she was put onto that new drug there was no communication back to her GP, and so when we went to the GP to have the first injection, I had to inform the GP of all of the specifics of that new drug and to almost reassure him that it was accurate, correct and efficacious before he would administer it. And rightly so, because he didn't have any information from the dermatology team warning him or giving him any information. And when the GP wrote to them to get information released, that wasn't released for about five months. So even when he asked for information, it wasn't given to him. (Consumer 57)*

### Ineffective collaborative practice outcomes

Ineffective collaborative practice led to consumers feeling disengaged and disempowered in their health care, resulting in negative impacts on their dignity and engagement in healthcare goals.

***Patient disempowerment.*** Consumers expressed being seen as another medical case rather than a person; consumers felt recognised by their condition rather than for their humanity. Healthcare professionals had not built relationships or considered consumers holistically, with consideration of emotional and psychological health needs in addition to their physical health needs.

*I nursed my dad while he was dying. He had cancer and we experienced some terrible care with dad, and I think the most memorable one was trying to get the nurses and the doctors to work together about inserting a catheter. And we'd gone into the emergency department in the evening and dad had not been able to pass urine sort since lunchtime and he was starting to get a pain… And I sort of said, you need to get a catheter for him. And they said, “Oh we need to get a doctor to check whether he needs a catheter”.… [about two hours later] after the doctor had come down and seen that he needed a catheter, she said to the nurse “Oh can you put the catheter in sister?”... And as a carer I felt so totally disempowered and unable to actually get for him what I as a non-trained medical person knew he needed. (Consumer 51)*

***Lack of coordinated healthcare goals.*** Ineffective collaborative practice contributed to negative impacts on coordinated patient care. Consumers were critical when witnessing health professionals dismiss invitations to work with other health professionals on shared healthcare goals, ultimately risking the patient achieving optimal health outcomes.

*I myself have had, for example, one specialist tell me that they can meet my health needs. But if I was to see them then they would no longer support me seeing my other specialist group because that would be just simply unnecessary despite the fact that my other specialist group deal with many and varied things that are far outside the scope of that particular specialist. They just simply didn't want the oversight by other health professionals which is certainly counter to a collaborative approach and quite frankly counter to getting a good outcome for the patient myself in that circumstance. (Consumer 59)*

Changing such an experience, would require a change in positioning of the consumer within their healthcare team. The themes underpinning this, as detailed by framework analysis, are explained below.

### Patient positioning within effective collaborative practice

Positioning within the healthcare team influenced the ability to deliver effective collaborative care. Positioning was described across all participant groups in relation to three sub-themes: patients as team members, family and carers as team members, and the role of power and hierarchy within the healthcare team.

***Patients as team members.*** There was consensus across all participant groups that collaborative practice was most effective, and patient centred when the patients’ lived experience, knowledge and priorities for their health were actively sought and prioritised. Consumers highlighted the importance of recognising the patient as an expert in their health experience, and the need to feel respected in order to communicate their preferences with the healthcare team. Consumers also acknowledged that the patient role in the team may vary depending on their level of knowledge, just as a health professionals’ role may vary depending on their clinical knowledge or experience.

*I think doctors and nurses and other health professionals come to a conversation thinking, especially newly graduated registrars or whoever it might be, that they have to know everything about the patient and they have to know what to tell the patient…. and I might know that already, but it might not give me time to ask questions…it's important to establish what the patient actually understands rather than just going off a script what you're supposed to impart to the patient. (Consumer 55)*
*Sometimes some patients would like …. handholding by the practitioner…they would like to be guided, they would like the practitioner to take the lead. But on the other hand, there are situations in which the consumer feels that they are very capable of deciding for themselves, then they would like to take the lead and want the practitioner to support them in making their decisions. So, it is important for practitioners to realise the different situations and play their role accordingly in a collaborative manner. (Consumer 60)*

Education providers spoke of the importance of patient centred collaboration being the focus in order to achieve the best health outcomes for the patient and their community.

*“So the collaboration is really all about a purpose. And the purpose, the collaboration is to achieve the best outcomes for our patients, the health outcomes for our community. So collaboration it’s got to have a purpose. It’s not just about sort of that we all get along in whether we’re all talking to each other, to achieve an outcome.” (Education provider 66)*

Health service representatives highlighted the role of recognising holistic care, giving consideration to the social determinants of health and having an overall understanding of what a patient needs when including the patient as a team member.

*I think the first thing is to recommend the patient centred care, to really look into how do we get this person better rather than I'm going there to do what I'm good at it, I'm gonna get this person better. So, to understand what the patient needs. (Health practitioner 63)*
*a collaborative practitioner should recognise that the health consumer is a sum of many equations and health is one component of what they seek. And the collaborative practitioners should recognise that the social determinants of health contribute to how they practice as well and how their patients would like them to practice. So apart from working within the sector, I think there needs to be more intersect collaboration as well as a holistic approach to how we interact and manage the patients. (Health practitioner 62)*

***Family, friends, carers and communities as team members.*** Consumers and education providers highlighted the importance of acknowledging the essential role and contributions of patients’ families, friends, carers and communities in their health care. Leveraging existing patient supports for shared goal setting and decision making were identified as key mechanisms for ensuring effective collaborative practice. It was also recognised that existing supports may play key roles in communicating important health information on behalf of the patient to other members of the health care team and vice versa.

*Health professions really need to catch up on co-design of care with people living with health issues and patient centred care and listening to patients and paying attention to what we are telling you about our experiences both as health consumers and as parents of kids with conditions and as carers and as families. So, we need to be at the centre of every single collaboration and of every single definition of collaborative healthcare. (Consumer 56)*
*You need someone who's going to be that linchpin, whether it's from the family or whether it's from the health workers from somewhere around the place who will be able to step in and will be able to provide all that information and communicate that information in a safe space and in an appropriate manner. (Consumer 54)*
*You need to think about that decision making process being a shared decision-making process with the patient and or their carers or family. (Education provider 83)*

***Power and hierarchy within healthcare teams.*** To achieve patient centred care, consumers and education providers spoke of goal setting and decision-making powers being shared across the entire team inclusive of patients. Consumers and education providers highlighted the need for health professionals to see their knowledge, skills, and roles as being complementary to others in a health care team. In some cases, this may result in someone in the health care team assuming a coordination or leading role to ensure shared goals are established in a holistic manner for a patient, that was also considerate of social determinants of health.

*I think if they didn't fight over turf and saw their services as complementary and sometimes in the community they play such an important role of coordination the pharmacists. I think that's something I have seen. (Consumer 51)*
*Collaboration also means for me that once communications established that they actually agree to work together to develop a real team approach to my care and developing a plan for my care that is genuinely holistic and is not just a piecemeal approach …that they're willing to make amendments and that they're willing to compromise as well. So, collaboration for me means and necessitates compromise that sometimes people are going to have to understand that maybe the particular treatment approach that they might want to take is not necessarily going to be the best one when you take it into consideration with all the other conditions that I have going on at the same time. (Consumer 56)*

Consumers emphasised how the patient was of equal importance in the health care team in the pursuit of holistic collaborative care. It was acknowledged by consumers and education providers that silos continue to exist in our health care sector. Perceived or actual professional hierarchies, where one health profession/professional may be seen or see themselves as having greater importance or value than others persist, risking the development of effective collaborative practitioners and teams. In the development of a collaborative practitioner and workforce, consumers and education providers acknowledged the need to flatten these perceived hierarchies and power differentials by embedding the philosophy of patient centred care and shared goal setting into health professions education.

*We need to be teaching from the get-go from university all the way through that this industry has no place for egos. You either treat everyone as an equal and you work together, or you are of little use to anyone in this industry. The patients first and foremost. (Consumer 59)*

### Effective collaborative practice outcomes

Positive narratives described elevated positioning of patients as well as their families and carers within the healthcare team and addressed existing or perceived silos and hierarchies across health professions. Patient empowerment and perception of shared goals resulted.

***Patient empowerment and optimism.*** Through acknowledging the important roles of patients, as well as their families, and addressing existing power differentials in the health care team, effective collaborative practice led to patient empowerment and optimism related to their health in the future, in addition to humanising the person being cared for.

*It was really encouraging. I felt supportive. I felt optimistic about my care. I felt optimistic about my long term prognosis, …. I felt like I had people who had my back. I felt like I had health professionals who I trusted and who had my best interests at heart, who were talking to each other, who were paying attention to all the symptoms that I was listing who were laying out quite clearly a plan for the management of my disease in 2019. (Consumer 56)*

***Shared goals for optimal patient outcomes.*** Effective collaborative practice was evident through the development of shared goals which were aligned with patients’ expressed goals as well as medically indicated needs, holistic, and agreed upon and communicated clearly to all healthcare team members.

*interprofessional is that idea of shared goals and really crystallising communication so that the patient is getting the same story from all of the team members (Education provider 23)*
*teamwork and thinking about the holistic needs of the patient is going to provide the best health outcomes for that patient. So learning how to utilise the people around you and working collaboratively with them is going to have the best health outcomes for your patient. (Education provider 23)*

## Discussion

Attributes of collaborative practice in Australia described in our findings are consistent with emerging as well as longstanding concerns previously identified in Australia ( Commonwealth of Australia, 2021; State of Victoria, 2021). There is a need to elevate the position of and prioritise listening to the voice of the patient as a member of the health care team, for improved health outcomes and efficiency. These concerns were echoed recently by Davidson *et al.* (2022) who point out that, even now, little is known about the patient role in a collaborative model of healthcare. Patient involvement in their care may take many forms and can change over time in response to the personal and clinical context ( Davidson *et al.*, 2022). For some, there is a desire to relinquish control to health professionals, others seek a more central role within the team by providing input to health professionals and taking a higher level of ownership for their care, while others choose to disengage from contributing ( Davidson *et al.*, 2022). Davidson emphasises that clinicians need to understand the role sought by patients at each stage of clinical interactions and adjust accordingly, and that the role probably stands on a continuum. Furthermore, the narratives confirmed that the person receiving care may readily disengage from their role due to a bad experience or when they have a challenge of another kind, e.g. moving home or transferring to a different sector of healthcare services. Thus challenges may remain for implementing effective collaborative practice.

Consumer narratives reiterate the frustration and challenge experienced in trying to navigate healthcare across a diverse clinical team. It seems remarkable that this discussion has been going on for almost 25 years, if not longer ( Towle *et al.*, 1999). Towle and colleagues attempted, through a comprehensive literature review, to define a series of competencies for health professionals that would eventuate in shared decision making. The first of these for the doctor was “develop a partnership with the patient” (p 766), and the authors were at pains to point out, even then, that “Informed shared decision making may also involve a team of health professionals, involve others (partners, family) and differ across cultural, social, and age groups.” (p766). Their first rule for the patient being cared for was “define (for oneself) the preferred Dr-Patient relationship” (p769). This view was not entirely without challenge or modification. Mariotto (1999) was perhaps the most far sighted in responding that “partnership is not a magic formula. The ground is not quite ready. Long term financial and cultural investment is required to realise this opportunity fully—but on the understanding that all patients have the right to delegate decisions to their doctor when this is the most comfortable solution for them” (p783). In addition, there seems to be an underlying issue of assumptions about consumer/patient health literacy in counter arguments to patient involvement: health practitioners not treating the patient as a team member because they assume they lack the literacy /capacity to take this role ( Campbell *et al.*, 2019). However, knowing that they can and are increasingly seeking to, should be a sufficient catalyst for change and a key consideration in the ongoing education and professional development of health professionals.

Nonetheless, the study clearly identifies the benefits, perceived by most participants, of a collaborative approach to healthcare. Additionally, the views of participants in this study also highlight the power differentials and hierarchy in our (in fact most) health care systems. This may relate to how individual professions are educated, but also to the prevailing culture in healthcare institutions. It also collides forcefully with the concept of scope of practice. Effectively, professional qualifications and training define what practitioners are permitted to do which is largely inherited from the parent profession ( Downie *et al.*, 2023). However there has been, in times of acute stress to health systems (e.g. COVID 19,) a loosening up of traditional scopes of practice, and sometimes a tightening (e.g. to prevent cross infection what tasks could be done were restricted). Central to the development of collaborative practice may be the idea that “no single profession holds exclusive ownership of a defined clinical knowledge, skill or task, and that many tasks can be safely and effectively performed by another professional group.” ( Downie *et al.*, 2023) (p 1195) A key message from the tone of the consumer narratives is that collaborative practice is not merely a set of technical skills and knowledge. It is achieved through health professionals’ behaviours, attitudes and values, rooted in professional standards and codes of conduct and also by the systems that drive health care in general. The impact of the Australian healthcare system on the ability to enact collaborative practice is described in a separate publication ( Kent *et al.*, 2024).

A limitation to this study was the breadth of participant sample; the perspective of only ten consumer advocates and four clinical practitioners was collected, largely due to the research being conducted during a period of COVID 19 disruption. Although the depth of the narratives collected was a strength, a broader and larger sample of participants, that included rural and underrepresented communities, would have been preferable.

## Ethics and consent

Ethics approval was granted by the Monash University Human Research Ethics Committee (ID: 34594, originally approved 05/09/2022, updated amendment approved 31/10/2022).

The explanatory statement and consent process was then communicated. All participants provided written informed consent prior to attending the focus group session.

## Data Availability

***Ethical and security consideration, Data protection issue*** Human data cannot be made available for this article. Ethical approval by the Monash University Human Research Ethics Committee (MUHREC) via the Participant Explanatory and Consent form limits sharing of raw data transcripts and specifically states: Focus group audio recordings will be destroyed once transcripts have been verified. Transcripts will be stored in a secure, password-protected shared folder only accessible by the research investigation team listed above. The data that support this study cannot be publicly shared due to ethical or privacy reasons and may be shared upon reasonable request to the corresponding author if appropriate. Requests can be submitted to: info@hpacf.org.au. All requests will be reviewed by the HPAC Forum IPE Working Group to ensure ethical or privacy considerations are not breached in accordance with the approvals provided by the Monash University Human Research Ethics Committee. Figshare: Methodological items: explanatory statements, consent forms, participant information, focus group question logics. https://doi.org/10.6084/m9.figshare.26342068 ( Meiklejohn, 2024). This project contains the following extended data: COMBINED EXPLANATORY STATEMENTS.pdf FG_QUESTIONS & RATIONALE_HEALTH SERVICES_FINAL-2.docx FG_QUESTIONS & RATIONALE_ED PROV_FINAL-2.docx FG_QUESTIONS & RATIONALE_CONSUMER_FINAL-2.docx FG_QUESTIONS & RATIONALE_CONSUMER_FINAL-2.docx PARTICIPANT INFORMATION.pdf COMBINED CONSENT FORMS.pdf Data are available under the terms of the Creative Commons Attribution 4.0 International license (CC-BY 4.0).
