# Supplementary material for: “The patients first and foremost” collaborative practice in the Australian healthcare system: a qualitative study
Source: MedEdPublish (2016). 2025 May 7;14:131. Originally published 2024 Sep 13. [Version 2] doi: 10.12688/mep.20512.2 (PMC12117322; doi:10.12688/mep.20512.2)
Supplement: Peer review history

# Reviewer report: Reviewer response for version 2

**DOI:** 10.21956/mep.22484.r41756

**Published:** 2025-05-27

**Authors:** Natalie Dodd

**Affiliations:** 1 University of the Sunshine Coast, Sippy Downs, Australia

The authors have strengthened the work, and I have no further comments. Well done team.

Have any limitations of the research been acknowledged?

No

Is the work clearly and accurately presented and does it cite the current literature?

Partly

If applicable, is the statistical analysis and its interpretation appropriate?

Yes

Are all the source data underlying the results available to ensure full reproducibility?

No source data required

Is the study design appropriate and is the work technically sound?

Yes

Are the conclusions drawn adequately supported by the results?

Partly

Are sufficient details of methods and analysis provided to allow replication by others?

Yes

Reviewer Expertise:

Communication skills, health professions education, interprofessional education, paramedicine

I confirm that I have read this submission and believe that I have an appropriate level of expertise to confirm that it is of an acceptable scientific standard.

# Reviewer report: Reviewer response for version 1

**DOI:** 10.21956/mep.21953.r38915

**Published:** 2024-10-07

**Authors:** Suyog Sindhu

**Affiliations:** 1 King George’s Medical University, Uttar Pradesh, India

The article examines collaborative patient-centered practice within the Australian healthcare system, a framework that remains poorly defined despite its significance. The research involved 19 focus groups with 84 participants, including education providers, consumers, representatives from the Health Profession’s Education Standing Group, and health service practitioners, conducted in 2022. Through framework analysis, the study aimed to identify the facilitators and barriers to collaborative practice in both education and healthcare settings.

 Participants highlighted several aspects of effective collaborative practice, emphasizing the necessity of incorporating the patient voice within healthcare teams. Central to the discussions was the role of patients, families, and carers as integral members of the healthcare team. The findings revealed that existing power dynamics and hierarchies within teams often hindered the implementation of collaborative practices.

 The study concluded that when patients and their families are actively involved in healthcare teams, it leads to shared goals and improved patient outcomes. Conversely, a lack of collaboration or exclusion of patients resulted in poor communication and fragmented care, ultimately causing patients to feel disempowered and disengaged. The article underscores the need for a clearer understanding and implementation of collaborative practices to enhance patient experiences in the healthcare system.

**Constructive Feedback:**

- **Quantitative Data**: Incorporating quantitative data or metrics to support qualitative findings could strengthen the conclusions. For instance, data on patient satisfaction or health outcomes could provide a more robust evaluation of collaborative practices.
- **Diversity of Perspectives**: While the focus groups included varied participants, expanding the scope to include more voices, such as those from rural or underrepresented communities, could provide additional insights into collaborative practice.
- **Recommendations for Implementation**: Offering specific recommendations for implementing collaborative practices based on the findings would provide actionable insights for practitioners and policymakers.
- **Discussion on Barriers**: A deeper exploration of the specific barriers identified in the study, perhaps with examples or case studies, could help contextualize the challenges faced in implementing collaborative practices.

Have any limitations of the research been acknowledged?

No

Is the work clearly and accurately presented and does it cite the current literature?

Yes

If applicable, is the statistical analysis and its interpretation appropriate?

Yes

Are all the source data underlying the results available to ensure full reproducibility?

Yes

Is the study design appropriate and is the work technically sound?

Yes

Are the conclusions drawn adequately supported by the results?

Yes

Are sufficient details of methods and analysis provided to allow replication by others?

Yes

Reviewer Expertise:

Interprofessional Education and Collaborative Practices

I confirm that I have read this submission and believe that I have an appropriate level of expertise to confirm that it is of an acceptable scientific standard.

# Reviewer report: Reviewer response for version 1

**DOI:** 10.21956/mep.21953.r38916

**Published:** 2024-10-07

**Authors:** Natalie Dodd

**Affiliations:** 1 University of the Sunshine Coast, Sippy Downs, Australia

Thank you for the opportunity to review this paper, a first of this online-style review for me. This is a clearly written manuscript that sought to describe collaborative practice in Australia, with the results being presented as examples of (or absence of) collaborative practice and the perceived or actual impacts these have on healthcare consumers. Please consider the following comments for potential improvements to this work.

**General comments**

 Title – ‘qualitative’ or ‘focus group’ should appear in the title (as per SRQR checklist).

 Checklist – I could not see a completed qualitative checklist (SRQR, COREQ) in the supplementary, nor could I find guidance around this on the peer-review portal, this is generally a requirement for most journals and may be worth considering completing and would provide guidance around a descriptive title.

**Introduction**

 Paragraph 1. I think this paragraph lacks focus with the first and second parts of the paragraph not closely relating to each other. The abstract background sentence ‘Collaborative patient centred practice is an expectation of the Australian healthcare system’ might be a better starting point to frame this important first interaction with the reader - The first four words clearly articulate what the whole paper is about.

 A definition of collaborative practice should be included here – perhaps the shared understanding describe in the focus group introduction (data collection). I can’t seem to find this working definition in the paper or the supplementary materials.

 I think there needs to be some discussion around interprofessional collaborative frameworks here, I’m not sure why these were omitted considering the focus of the paper. The updated Canadian Interprofessional Health Collaborative  Competency Framework  for Advancing Collaboration (2024) which places the patient at the centre of the collaborative framework is worth considering https://cihc-cpis.com/wp-content/uploads/2024/06/CIHC-Competency-Framework.pdf

 Other frameworks may also be relevant here.

 Paragraph 2. “In some settings, healthcare is provided by interprofessional teams and care has been associated with improved patient outcomes”, some examples from the literature cited should be included here.

 There is some newer data about interprofessional learning in multidisciplinary teams and the impact on patient outcomes that may be worth considering for inclusion here, Webster et al., 2024 https://pubmed.ncbi.nlm.nih.gov/37921751/

**Methods**

 Study design – well described

 Data collection – I realise you include this in results, but on first read through I was wondering if the focus groups were a mix of participant types or not. It might help the reader of this is clarified in this section.

 I think there needs to be some clarification that there were three separate focus group questions sets early on in this section.

 Data analysis – “It is however recognised that the term ‘person-centred care’ and  other alternatives have been adopted as a preferred term elsewhere” requires citation/s

**Results**

 Consider removal of duplication of content describing participants, the following matches the data within table 1.

 “This included two consumer focus groups (n=10 participants), fourteen education provider focus groups (n=62 participants), two health practitioner focus groups (n = 4 participants),  and one focus group was comprised of representatives from the Health Professions Education Steering Group, of Universities Australia a national peak body representing 39 Australian  universities (n=8 participants)”

 Minor formatting issue. All quotes should commence with either a capital letter or lower case letter, not a combination of both.

 Patient disempowerment. I think this quote reflects patient-centered communication more so than collaboration more broadly. The lack of time to communicate prevents the development of rapport and trust. Given that the competencies of collaborative practice are not a focus of this paper, I think more needs to be done to relate this comment to the aim.

 Lack of coordinated healthcare goals – this is an important and well written finding.

**Discussion**

 “Attributes of collaborative practice in Australia described in our findings are consistent with emerging as well as longstanding concerns previously identified in the published literature” requires citations.

 This quote “For some, there is a desire to relinquish control  to health professionals, others seek a more central role within the team by providing input to health professionals and taking a higher level of ownership for their care, while others  choose to disengage from contributing” highlights why the introduction may need a shift in focus as this contradicts the first sentence of this paper, i.e., a patient-centred approach may not mean ‘active participation of patients’.

 “health practitioners not treating the patient as a team member because they assume they lack the literacy /capacity to take this role” requires citation

 “Another aspect of this large study includes articulation of the competencies required of all health professionals, to better facilitate the enactment and experience of collaborative care (Kent et al., 2023a)” does not seem to fit here, this should perhaps be moved to a strengths and weaknesses section, and listed as a strength.

 “(e.g. to prevent cross infection what tasks could be done were restricted)” needs to be reworded.

 “It is achieved through health professionals’ behaviours, attitudes and values”, this claim could be strengthened by reference to professional capability documents, codes of conduct etc.

Have any limitations of the research been acknowledged?

No

Is the work clearly and accurately presented and does it cite the current literature?

Partly

If applicable, is the statistical analysis and its interpretation appropriate?

Yes

Are all the source data underlying the results available to ensure full reproducibility?

No source data required

Is the study design appropriate and is the work technically sound?

Yes

Are the conclusions drawn adequately supported by the results?

Partly

Are sufficient details of methods and analysis provided to allow replication by others?

Yes

Reviewer Expertise:

Communication skills, health professions education, interprofessional education, paramedicine

I confirm that I have read this submission and believe that I have an appropriate level of expertise to confirm that it is of an acceptable scientific standard, however I have significant reservations, as outlined above.
